# Charcot Marie Tooth disease (CMT4A) due to GDAP1 mutation: report of a Colombian family

**Published:** 2015-12-30

**Authors:** Angela M Martin, Silvia J Maradei, Harvy M Velasco

**Affiliations:** School of Medicine. Instituto de Genética, Universidad Nacional de Colombia, Bogota, Colombia.

**Keywords:** Founder effect, hereditary sensory and motor neuropathy, axonal neuropathy, genetic counseling, Charcot-Marie-Tooth Disease

## Abstract

**Background:**

Mutations of *GDAP1* gene cause autosomal dominant and autosomal recessive Charcot-Marie-Tooth disease and more than 40 different mutations have been reported. The recessive Q163X mutation has been described in patients of Spanish ancestry, and a founder mutation in South American patients, originating in Spain has been demonstrated.

**Objective:**

We describe physical and histological features, and the molecular impact of mutation Q163X in a Colombian family.

**Methods:**

We report two female patients, daughters of consanguineous parents, with onset of symptoms within the first two years of life, developing severe functional impairment, without evidence of dysmorphic features, hoarseness or diaphragmatic paralysis. Electrophysiology tests showed a sensory and motor neuropathy with axonal pattern. Sequencing of *GDAP1* gene was requested and the study identified a homozygous point mutation (c.487 C>T) in exon 4, resulting in a premature stop codon (p.Q163X). This result confirms the diagnosis of Charcot-Marie-Tooth disease, type 4A.

**Results:**

The patients were referred to Physical Medicine and Rehabilitation service, in order to be evaluated for ambulation assistance. They have been followed by Pulmonology service, for pulmonary function assessment and diaphragmatic paralysis evaluation. Genetic counseling was offered. The study of the genealogy of the patient, phenotypic features, and electrophysiological findings must be included as valuable tools in the clinical approach of the patient with Charcot-Marie-Tooth disease, in order to define a causative mutation. In patients of South American origin, the presence of *GDAP1* gene mutations should be considered, especially the Q163X mutation, as the cause of CMT4A disease.

## Abstract (es)

**Antecedentes:**

Las mutaciones del gen *GDAP1* son causantes de la enfermedad de Charcot Marie Tooth tanto autosómica dominante como recesiva, y se han reportado más de 40 mutaciones distintas. La mutación recesiva Q163X ha sido descrita en pacientes de ascendencia española y se ha demostrado una mutación fundadora originaria de España en pacientes de origen suramericano. Describimos las características físicas e histológicas y el impacto molecular de la mutación Q163X en una familia colombiana.

**Objetivo:**

Se describe el impacto de la mutación Q163X en las características físicas, histológicas y moleculares en una familia colombiana.

**Métodos:**

Se describe dos pacientes de sexo femenino, hijas de padres consanguíneos, quienes presentaron inicio de síntomas en los dos primeros años de vida, mostrando deterioro funcional severo, sin evidencia de dismorfía, disfonía o parálisis diafragmática**. **Los estudios de electrofisiología mostraron una neuropatía sensitiva y motora con patrón axonal. Se solicitó la secuenciación del gen *GDAP1*, y el estudio identificó una mutación homocigota puntual (c. 487 C>T) en el exón 4, causando un codón de parada prematuro (p. Q163X). Este resultado confirma el diagnóstico de Enfermedad de Charcot Marie Tooth, tipo 4A (recesiva, tipo axonal).

**Resultados:**

Las pacientes fueron remitidas al servicio de Fisiatría para evaluación de métodos de asistencia para deambulación. Ellas reciben seguimiento por el servicio de Neumología, quienes vigilan la función pulmonar y el desarrollo de parálisis diafragmática. Se brindó asesoramiento genético. La genealogía del paciente, las características fenotípicas y los hallazgos en los estudios electrofisiológicos son herramientas valiosas en el enfoque clínico del paciente con CMT, de forma que se pueda plantear una posible mutación causal. Se debe considerar la presencia de mutaciones en el gen *GDAP1* en pacientes de origen suramericano, en especial la mutación Q163X, como causa de CMT4A.

## Introduction

Charcot Marie Tooth (CMT) disease, also known as Hereditary Motor and Sensory Neuropathy (HMSN) refers to a group of disorders characterized by a chronic motor and sensory polyneuropathy. It is the most frequent inherited peripheral neuropathy, and the most common inherited pathology of the nervous system, with a prevalence of 1 in 2,500 people 1. There is no prevalence difference in gender or ethnicity regarding disease prevalence, and the age of presentation varies depending on the type of CMT disease.

The hereditary neuropathies are classified into several categories, based on their type of inheritance, age of onset and characteristics of nerve conduction 2. Charcot Marie Tooth 1 (HMSN type I according to Dyck's classification), comprises the group of demyelinating peripheral neuropathies and CMT2 (HMSN type II) comprises the axonal peripheral neuropathies 3. Both groups share an autosomal dominant inheritance pattern, which is the most frequent mode of inheritance in CMT disease 4. Charcot Marie Tooth 4 (Autosomal-recessive HMSN) refers to the neuropathies with an autosomal recessive inheritance pattern, and includes both demyelinating and axonal types.

 Charcot Marie Tooth 4A is the most common subtype of CMT4 2. The only gene associated with CMT4A is *GDAP1* 2,4, which codifies for the Ganglioside-induced differentiation-associated protein 1, a glutathione transferase. There have been described more than 40 disease-causing mutations of *GDAP1* gene, both dominant and recessive 1, and one of the most frequent is Q163X mutation. This mutation has been described as a founder mutation on patients with Spanish ancestry, and its origin was tracked to the region of Castilla, Lion and Basque Country in Spain 5.

Here we present two female patients, daughters of consanguineous parents, with an early onset of symptoms, and an axonal neuropathy in whom a *GDAP1* Q163X mutation was found. This is the first case of autosomal recessive CMT reported in our country, and also the first reported case of a GDAP1 mutation.

Here we make a review of literature, describing the features of the *GDAP1* gene, especially the Q163X mutation, and its implications in the development, diagnosis and prognosis of CMT4A.

## Case description

### Patient 1 

**Figure 1.**
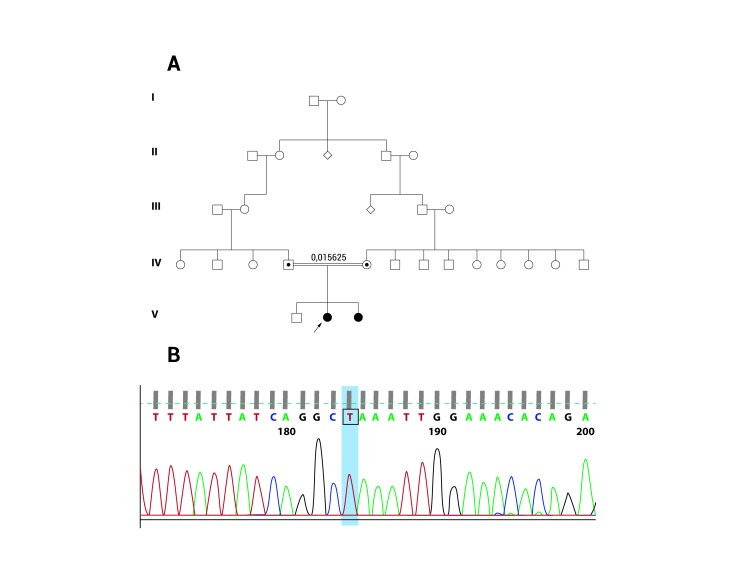
**A**. Case 1 Patient. **B**. Case 2 Patient. Patient. Both patients developed similar symptoms. Note the severe and symmetric muscle hypotrophy of limbs, contracture deformities of the fingers and elbows, marked atrophy of tenar, hypotenar and interosseous muscles of the hands*, pes cavus*, without dysmorphic features.

A 14-year-old female was referred to our consultation, with a progressive deformity in valgus of inferior limbs and muscle hypotrophy (Fig. 1a). The onset was at 15 months old. She gradually developed gait instability, reduced range of motion on hands and arms, and impaired gross and fine motor skills. She is the second child of a mother Gravida 3 Para 3, and healthy consanguineous parents. The pregnancy was uneventful, vaginal delivery at 39 weeks, with birth weight 2,600 g (5th centile), and birth length 45 cm (25th centile). Developmental milestones were normal, with sitting at 6 months, and walking at 15 months of age. Intelligence is normal, with overall good academic performance. At general examination, she was a wheelchair bound but otherwise healthy appearing girl. Her weight was 25 kg (3rd centile), her height was 132 cm (below 3rd centile), and her head circumference was 52 cm (16th centile). At physical examination, diminished sensitivity on hands, forearms, feet and calves, scoliosis and pes cavus were found. Muscle atrophy was evident, predominantly on forearms and calves, with contractures of fingers. Dysmorphic features were not seen, vocal paresis and breathing difficulty were notably absent. Achillean and bicipital reflexes were diminished, superior limbs showed weakness 4/5 on hands, 4/5 on forearms, and 4/5 on shoulder girdle (according to the MRC scale). Inferior limbs showed weakness 3/5 on thighs, calves and feet. The cranial nerves were unaffected, and dysautonomic signs were not found.

The brain MRI was normal, as well as liver enzymes. Motor nerve and sensory nerve conductions (NCV) of the patient were absent in all explored nerves. EMG (electromyography) of the lower extremities showed normal insertional activity in all explored muscles; abnormal spontaneous activity was present in right rectus femoris; increased amplitude and reduced recruitment were present in left anterior tibialis, left gastrocnemius and left rectus femoris.

In conclusion, the NCV and EMG studies showed a sensory and motor neuropathy, axonal type, with signs of reinnervation, consistent with a chronic process and severe expression.

**Figure 2.**
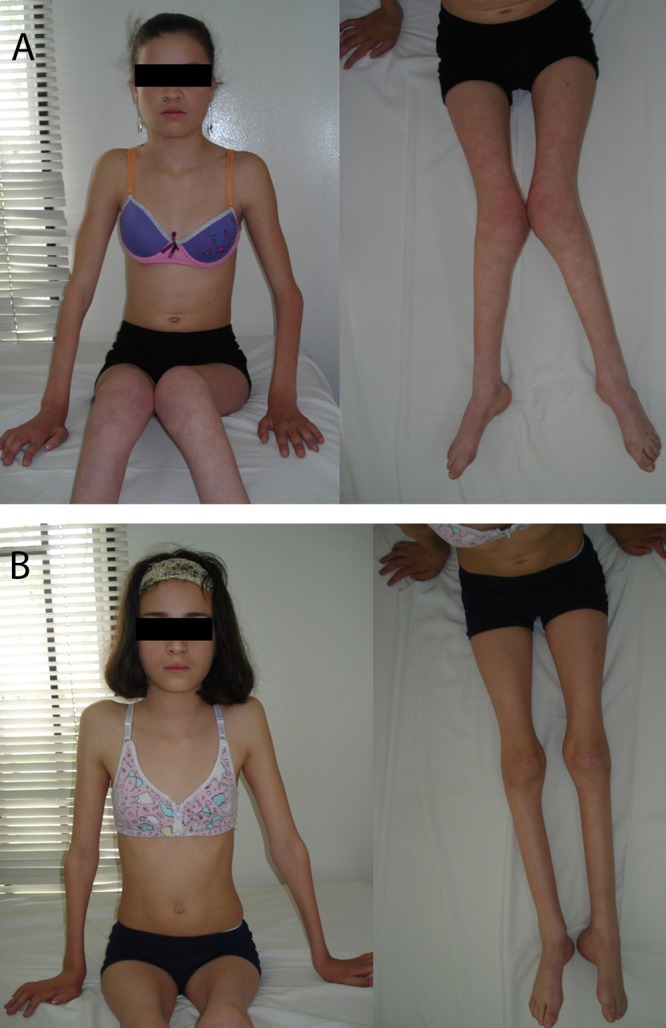
Family tree of the patients. **A**. Arrow shows patient Case 1. Older sibling is unaffected; however, genetic testing has not been done because of the age of the patient. There are no other known affected members, and carrier status is not available for other family members. **B**. DNA sequencing results of the patient Case 1, showing Q163X mutation at *GDAP1* gene (c.487 C>T).

Sequencing of *GDAP1* gene was requested in this patient. The study identified a homozygous point mutation (c.487 C>T) in exon 4, resulting in a premature stop codon (p.Q163X) (Fig. 2). This result confirms the diagnosis of Charcot-Marie-Tooth disease, type 4A (recessive, axonal type).

### Patient 2 

This 10-year-old patient is the younger sister of the first patient described above (Fig. 1b). She was referred to our service, with a progressive deformity in valgus of inferior limbs, impaired gait and multiple falls. The onset of symptoms was at 15 months old. At 3 yrs of age she developed progressive muscle hypotrophy, and reduced range of motion in hands at 4 yrs of age. She is the third child of consanguineous parents, with an uneventful pregnancy, vaginal delivery at 40 weeks, with birth weight 2,600 g (5th centile) and birth height 46 cm (25th-50th centile). She developed neonatal hyperbilirubinemia, and received phototherapy. Developmental milestones were normal, with sitting at 7 months, and walking at 12 months of age. Intelligence is normal. On general examination, she required crutches, but remained ambulatory. Otherwise she was a healthy appearing girl. Her weight was 22 kg (5th centile), her height was 129 cm (between 10th and 25th centile), and her head circumference was 53 cm (50th centile).

At physical examination, there was muscle atrophy in thenar and hypothenar areas of the palm, and interosseus muscles in both hands, as well as in lower limbs. Pes cavus was present. Achillean and bicipital reflexes were absent. Weakness three-fifths in feet, calves and tights, and weakness four-fifths in hands, forearms and shoulder girdle were found (according to the MRC scale). Dysmorphic features were not seen. Vocal cords paresis and breathing difficulty were absent.

Neuroconduction velocities were absent in all explored nerves. EMG of the upper and lower extremities showed normal insertional activity in all explored muscles; without abnormal spontaneous activity. Reduced recruitment was seen in all explored muscles. Increased amplitude with polyphasic morphology was also seen in right anterior tibialis and left biceps muscles.

In conclusion, the NCV and EMG studies showed a sensory and motor neuropathy, axonal type, with signs of reinnervation.

## Discussion

The review made by Yiu and Ryan 6 gives a useful methodology for the diagnostic approach of the child with neuropathy. Unlike in adult, in whom the main cause of neuropathy is diabetes, in children is genetic disease. Its clinical features are delayed motor milestones (when the neuropathy develops in the first year of life), proximal weakness, foot deformity or scoliosis. In these children it must be evaluated whether the neuropathy is associated with a central nervous disorder, whether the neuropathy is axonal or demyelinating, the inheritance pattern, and distinguishing features (dysmorphic traits, mental retardation, and other neuropsychological manifestations).

The patients had a disease onset the two first years of life, with progressive and symmetric hypotrophy, gait instability and foot deformity, at the time they had already completed the milestones according to their age. Both of them showed similar disease development, and none of them had dysmorphic features or mental retardation. These findings, in the context of a neuropathy, guided the authors to the diagnosis of a hereditary sensory and motor neuropathy.

The EMG and NCV showed a severe axonal neuropathy in both patients, suggesting the diagnosis of Charcot-Marie-Tooth disease. We made a review of the patients' pedigree (Fig. 2). The parents are first grade cousins, both of them with no relevant medical history, and there are no other affected members. The patients have an otherwise healthy older brother. The pattern of inheritance showed in the pedigree suggest an autosomal recessive disease 7.

We followed the algorithm developed by England *et al*. 8, on the basis of a positive familiar history (two members affected), an axonal neuropathy found in the patients, age of onset, and an autosomal recessive pattern of inheritance. Sequencing of *GDAP1* gene was requested in the first patient.

The nerve biopsy is reserved for patients in whom genetic test does not yield to a molecular diagnosis, with an atypical presentation or in whom inflammatory neuropathy is suspected 9. None of these features were found on the patients.*GDAP1* gene sequencing showed a homozygous point mutation (p.Q163X), which led us to the diagnosis of Charcot-Marie-Tooth disease, type 4A. In consequence, nerve biopsy was not requested.

 The mode of inheritance is autosomal recessive. Both parents are obligate carriers of the mutation, and are not at risk of developing the disease. Siblings of the parents have a 50% risk of being a carrier. After obtaining the genetic test result, the parents of the patients were counseled about the disease.

The older brother was described as normal by the parents of the patients. Development milestones were normal, and he shows no signs of neuropathy or mental retardation. Phenotype and physical examination are otherwise normal. In this case, we consider the brother as unaffected, and as a sibling of an affected individual, he has a two-thirds chance of being a carrier. The decision to undergo genetic testing must be done by the patient himself, when he reaches the age of majority. In the case of being a carrier, genetic counseling must be offered, as well as prenatal and/or preimplantation genetic testing, in order to discuss reproductive options and risks of the offspring.

## Features of GDAP1 protein

The *GDAP1* gene locus is situated on chromosome 8q13-q21. The protein is located in the outer mitochondrial membrane 1 as an integral membrane protein 10. It is mainly found in neuronal cells of peripheral and central nervous system 1, but also is expressed by myelinating Schwann cells of the peripheral nervous system 10. GDAP1 increases cellular glutathione-s-transferase in neuronal cells, decreases reactive oxygen species production, protecting against oxidative stress 1. GDAP1 has also a function on the balance between fused and fragmented mitochondria 10, which seems to be essential for the integrity of myelinated peripheral nerves, and it is also a peroxisomal fission factor: lack of GDAP1 cause altered peroxisomal morphologies, overexpression promotes peroxisomal fragmentation 11.

More than 40 disease-caused mutations in the *GDAP1* gene have been identified 1. Demyelinizing (R161H), axonal (S194X) and intermediate (T117fs) patterns on NCV can be found, as well as autosomal dominant (R120W, T157P, Q218E, C240Y) 12 or recessive (Q163X) patterns of inheritance; a wide spectrum of clinical features are seen, and the course of the disease is different between each type of *GDAP1* mutation. Dominant *GDAP1* mutations are associated with mild axonal neuropathy with late onset and slow disease progression, the extent and severity of proximal weakness is less pronounced compared with recessive mutations, and the electrophysiology findings are variable 12,13. Recessive *GDAP1* mutations have a more severe phenotype and an early onset, before age 10; vocal cord paresis, facial weakness and diaphragmatic paralysis are common features 12.

The Q163X (c.487 C>T) mutation is one of the most common recessive mutation on *GDAP1* gene 12. This mutation is located in exon 4, and results in a premature stop codon that affects the GST domain 14. Niemann *et al*. 10, showed that the GDAP1 mutated protein was not detectable in transfected cells, suggesting that the protein is highly unstable, and the mutation behaves as a null mutation.

Cuesta *et al*.^15^, reported three different families with Spanish ancestry, in which the mutation Q163X was present in the affected individuals as a homozygous form, or a compound heterozygous with S194X mutation. Boerkoel *et al*. 16, reported five Hispanic patients with a homozygous Q163X mutation, four of them from Costa Rica, and one from Peru.

Claramunt *et al*. 5, demonstrated that the Q163X is the most frequent mutation in Spain, with an estimated age of 33,000 years old (1,650 generations, 20 year generations). They made a comparison between haplotypes of patients with Q163X mutation from Castile and Leon, Basque Country, and the Region of Valencia, the patients reported by Cuesta *et al*. 15, and the haplotypes of the patients described by Boerkoel *et al*. 16, and confirmed that the mutations found in the patients have a common ancestral origin. This suggests the existence of a founder effect. In Colombia, this mutation has not been previously described. It might be possible that this mutation is also caused by the same founder mutation described previously, however, more studies are necessary to confirm this hypothesis.

Although the patients with a Q163X mutation show heterogeneous phenotypes, even within the same family, they share some features that differentiate the CMT4A caused by a GDAP1 Q163X mutation from other forms of CMT. The main phenotype features described of the patients with the *GDAP1* Q163X mutation 15 are, a clinical onset in childhood with weakness and foot and hand wasting, an axonal pattern on nerve conduction velocities, hoarse voice and vocal cord paresis with onset in the second decade of life, and normal intelligence. Pes cavus, claw hands and contractures were also seen. Most of them were wheelchair bound after 10 years of age.

Other authors 16,17 have described a phenotype defined by an early onset of symptoms, usually during the first year of life, consisting in progressive distal muscle weakness, sensitive loss, steppage gait, pes cavus, kyphoscoliosis, hammertoes, and reduced or absent reflexes. Patients are wheelchair bound by the second decade of life. Developmental milestones are achieved on time prior to the onset of disease, and the intelligence is always preserved.

The patients of this case description showed almost all the clinical features described on these case reports. Hoarse voice, vocal cord paresis and diaphragmatic paralysis were absent at the time of the submission of this paper. The spirometry was normal in both patients, suggesting no vocal or diaphragmatic compromise.

The NCV and EMG patterns in the patients with Q163X mutation are heterogeneous. Boerkoel *et al*. 16, reported undetectable median nerve motor conduction velocities in 4 of five patients, and undetectable median nerve sensory conduction velocities in 2 of five patients. Claramunt *et al*. 5, reported nerve conduction velocities (NCV) between 24 and 45 m/s. The patients reported by Sevilla *et al*. 17, had NCVs above 40 m/s, and patients without report due to severe distal muscular atrophy, had conserved proximal motor latencies. In both of our patients, EMG showed an axonal pattern; on the other hand, the NCV were absent in all of the explored muscles. The advanced stage of disease, and the severe hypotrophy might have caused these findings in the patients.

Histopathological findings associated with Q163X mutation include severe loss of myelinated axons, moderate loss of unmyelinated fibers, and onion bulb formations, with no evidence of axonal renewal 16. Onion bulb formations are less evident in compound heterozygous patients 17. 

##  Conclusion

The study of the genealogy of the patient, the phenotype features, and the NCV and EMG findings must be included as valuable tools in the clinical approach of the patient with Charcot-Marie-Tooth disease, recessive axonal type. In patients of South American origin, it should be considered the presence of *GDAP1* gene mutations, especially the Q163X mutation, as the cause of CMT4A disease. More information of the genetic background of CMT4A disease in Colombia and other South American countries is needed, in order to describe it, and understand its clinical course and prognosis.
